# Developing a model for rehabilitation in the home as hospital substitution for patients requiring reconditioning: a Delphi survey in Australia

**DOI:** 10.1186/s12913-023-09068-5

**Published:** 2023-02-03

**Authors:** Roslyn G Poulos, Andrew M Cole, Kerry N Warner, Steven G Faux, Tuan-Anh Nguyen, Friedbert Kohler, Fey-Ching Un, Tara Alexander, Jacquelin T Capell, Dan R Hilvert, Claire MC O’Connor, Christopher J Poulos

**Affiliations:** 1 HammondCare, Sydney, Australia; 2 School of Population Health, UNSW, Sydney, Australia; 3 St Vincent’s Hospital, Sydney, Australia; 4 South Western Sydney Local Health District, Sydney, Australia; 5 Australasian Rehabilitation Outcomes Centre, University of Wollongong, Wollongong, Australia; 6 Hilvert Advisory, Sydney, Australia

**Keywords:** Rehabilitation, Rehabilitation in the home, Reconditioning, Delphi study, Allied health, Community rehabilitation, Post-acute rehabilitation

## Abstract

**Background:**

Reconditioning for patients who have experienced functional decline following medical illness, surgery or treatment for cancer accounts for approximately 26% of all reported inpatient rehabilitation episodes in Australia. Rehabilitation in the home (RITH) has the potential to offer a cost-effective, high-quality alternative for appropriate patients, helping to reduce pressure on the acute care sector. This study sought to gain consensus on a model for RITH as hospital substitution for patients requiring reconditioning.

**Methods:**

A multidisciplinary group of health professionals working in the rehabilitation field was identified from across Australia and invited to participate in a three-round online Delphi survey. Survey items followed the patient journey, and also included items on practitioner roles, clinical governance, and budgetary considerations. Survey items mostly comprised statements seeking agreement on 5-point Likert scales (strongly agree to strongly disagree). Free text boxes allowed participants to qualify item answers or make comments. Analysis of quantitative data used descriptive statistics; qualitative data informed question content in subsequent survey rounds or were used in understanding item responses.

**Results:**

One-hundred and ninety-eight health professionals received an invitation to participate. Of these, 131/198 (66%) completed round 1, 101/131 (77%) completed round 2, and 78/101 (77%) completed round 3. Consensus (defined as ≥ 70% agreement or disagreement) was achieved on over 130 statements. These related to the RITH patient journey (including patient assessment and development of the care plan, case management and program provision, and patient and program outcomes); clinical governance and budgetary considerations; and included items for initial patient screening, patient eligibility and case manager roles. A consensus-based model for RITH was developed, comprising five key steps and the actions within each.

**Conclusions:**

Strong support amongst survey participants was found for RITH as hospital substitution to be widely available for appropriate patients needing reconditioning. Supportive legislative and payment systems, mechanisms that allow for the integration of primary care, and appropriate clinical governance frameworks for RITH are required, if broad implementation is to be achieved. Studies comparing clinical outcomes and cost–benefit of RITH to inpatient rehabilitation for patients requiring reconditioning are also needed.

**Supplementary Information:**

The online version contains supplementary material available at 10.1186/s12913-023-09068-5.

## Background

Reconditioning for patients who have experienced functional decline following medical illness, surgery or treatment for cancer is a growing inpatient rehabilitation impairment category in Australia. Defined as rehabilitation for ‘generalised deconditioning not attributable to any of the other impairment groups’ such as stroke, cardiac disorders, orthopaedic disorders, pain disorders, or neurologic conditions (see Australasian Rehabilitation Outcomes Centre (AROC) impairment codes 16.1, 16.2 and 16.3 [1]), the number of episodes for reconditioning rehabilitation has doubled in volume within a decade, increasing from 16,120 episodes in 2010 [2] to 32,877 in 2019 [3]. With 26.2% of all inpatient rehabilitation episodes reported to the national integrated outcomes centre (AROC) being for reconditioning [4], this is the largest inpatient rehabilitation impairment type in Australia.

There is growing evidence for the effectiveness of inpatient rehabilitation for reconditioning (e.g.,[5–7]; and data on inpatient rehabilitation episodes in Australia show improvement in Functional Independence Measure (FIM)[8] scores across the six Australian case-mix funded rehabilitation classes that involve reconditioning (Australian National Subacute and Non-Acute Patient (AN-SNAP) classes 4AR1 through 4AR6 [3]). However, rehabilitation services are challenged by increasing demand [9, 10], and patients requiring rehabilitation spend on average 12% of their acute hospital admission waiting for a rehabilitation bed [11].

Ambulatory rehabilitation models of care have been shown to be effective for a range of conditions when appropriate patient selection is undertaken (see for example, uncomplicated total knee arthroplasty [12] and early supported discharge services for stroke [13]). Nevertheless, given the substantial heterogeneity amongst the reconditioning patient group, it is important to give careful consideration to defining the characteristics of those who can be safely and cost-effectively managed at home.

While there are examples of ambulatory rehabilitation in Australia [14], it is unclear how many of these services provide RITH as hospital substitution for reconditioning. To our knowledge, there are no published studies or models on RITH as hospital substitution for this impairment category; however, RITH has the potential to offer a cost-effective and high-quality alternative to inpatient care. Home is where patients may prefer to recover, and the home environment is more contextually relevant for the patient, thus potentially improving the patient’s rehabilitation experience [15, 16]. Increasing access to RITH for reconditioning may also reduce pressure on the acute care sector if it allows greater rehabilitation capacity. Further, the acute care burden of the COVID-19 pandemic has highlighted the need for responsive health services, with a greater focus on in-home care. COVID-19 has also brought its own rehabilitation/reconditioning challenges that will likely include the need for expanded RITH options [17, 18].

Thus, in order to consider options to address the increasing demand for reconditioning following acute hospitalisation, this study sought to gain consensus on key elements for a model for RITH as hospital substitution for reconditioning. This paper describes a Delphi process with panellists from across Australia and reports on a consensus driven model for RITH.

## Methods

This research was led by a multidisciplinary project team of academics (*n* = 4), rehabilitation physicians (*n* = 6), allied health professional (*n* = 1), and a health service financing consultant (*n* = 1), during 2021/22. A three-round Delphi survey [19] was used to explore, refine and seek consensus on aspects of RITH as hospital substitution for patients requiring reconditioning following medical illness, surgery or treatment for cancer (AROC impairment codes 16.1, 16.2 and 16.3 [1]).

For the Delphi survey, a multidisciplinary group of health professionals working in the field of rehabilitation was identified from across Australia in one of two ways. First, the project team identified health professionals with knowledge, skills and experience in the field of rehabilitation, known to them from within their professional community, publications or conference presentations. Each identified professional received a personal email invitation from a project team member informing them of the upcoming survey. Second, to broaden the sample, other participants were sought through an emailed news item to the membership of AROC, informing them about the study and inviting individuals with experience in rehabilitation for reconditioning and/or rehabilitation in the home (from medicine, allied health, nursing and health service management) to express their interest in the survey by contacting the research fellow. The membership of AROC comprises 250 public and private rehabilitation services (almost all inpatient rehabilitation services) across Australia.

All potential Delphi participants (those personally invited and those AROC-sourced) then received an individualised link to the online survey (developed on the Qualtrics platform[20]), along with a participant information sheet (PIS). Access to the survey questions was only enabled after a participant selected the option which acknowledged that they had read the PIS and were providing their consent to participate. Only those who responded to the first Delphi round (DR1) were invited to the second round (DR2); and only participants from the second round were invited to the third round (DR3). Each round was open for two weeks, during which two email reminders were sent.

**Fig. 1:**
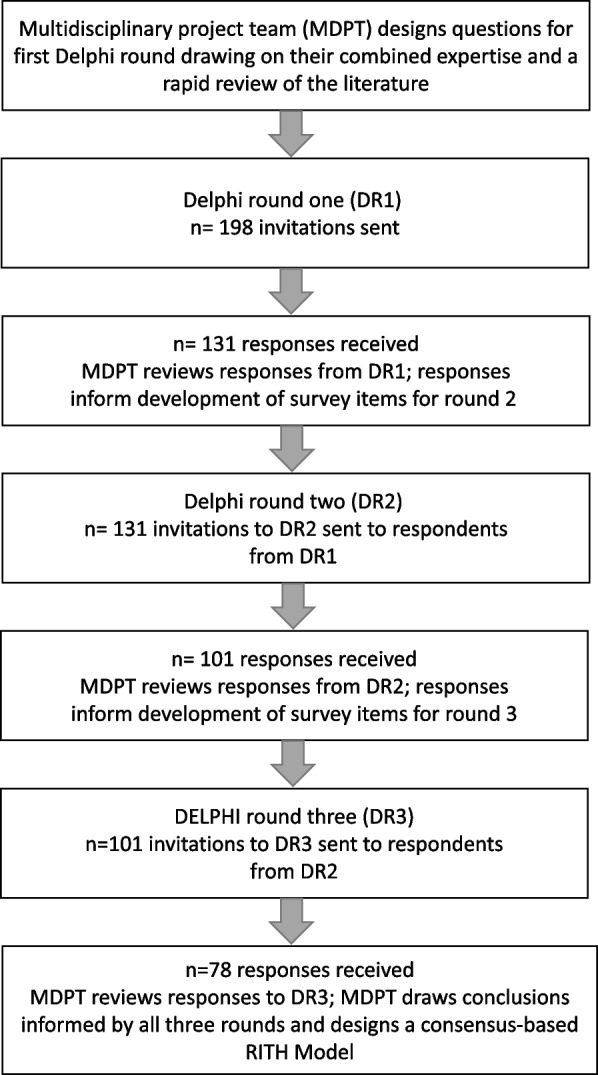
Delphi study flow chart

DR1 was developed by the project team, drawing on their combined expertise from working and researching in the rehabilitation setting, and from a rapid review of the literature on home-based rehabilitation services which followed an approach informed by Haby et al. [21] and Pandor et al. [22]. The rapid review of the literature identified areas where more information or additional clarity was required, thus informing the breadth and content of the Delphi survey. Survey items were grouped into categories which roughly followed the patient journey. Categories included: initial patient identification for RITH; determination of patient eligibility; patient assessment and development of the RITH care plan; case management and RITH program provision; patient and program outcomes; as well as practitioner roles (general practitioner (GP), rehabilitation physician), clinical governance and budgetary considerations. Data from DR1 and DR2 were reviewed and informed the project team's development of survey items for subsequent rounds. DR2 and DR3 survey items included only issues for which consensus had not been achieved in a previous round, or tested issues within the survey categories that participants had raised in free text boxes. See Fig. 1.

Survey items mostly comprised statements seeking agreement on 5-point Likert scales (from *strongly agree* to *strongly disagree*). A priori, it was decided that consensus on these items was reached when at least 70% of participants either *agreed*/*strongly agreed* or *disagreed*/*strongly disagreed* with a proposed statement. A small number of questions required participants to rank items in order of preference (DR1), choose one item from a list (DR2), or estimate percentages (DR3). At the end of each category a free text box was provided, allowing participants an opportunity to qualify item answers or make comments. Responses from DR1 and DR2 were broadly summarised and shared with participants in DR2 and DR3, respectively.

## Data analysis

Descriptive analysis was undertaken for participant characteristics and group responses to survey items using SPSS V27. Qualitative written statements were carefully read, and new or untested issues were noted, discussed with the project team, and informed questions in the subsequent round where necessary. Qualitative written statements were also used in understanding item responses.

## Results

Personalised email invitations from the project team were sent to 129 professionals; 69 other professionals expressed an interest in the survey in response to the AROC news item and subsequently received a survey invitation through Qualtrics. Overall, 131 professionals of the 198 receiving an invitation participated in DR1, giving a response rate of 66% (comprising 79 participants from personal invitation, and 52 participants from those who had responded to the AROC news item). Response rates to both DR2 and DR3 were 77% (101/131 and 78/101, respectively).

A broad range of disciplines (see Table 1) and representation from most Australian states was achieved across the three rounds (DR1 to DR3: New South Wales 56.5%—50.0%, Victoria 19.8%—24.4%, Queensland 8.4%—9.0%, South Australia 6.9%—7.7%, Western Australia 5.3%—5.1%, missing data 3.1 – 3.9%).

**Table 1:** Distribution of participants across the three Delphi rounds

	Round 1 (DR1)	Round 2 (DR2)	Round 3 (DR3)
Administrator-health services	6 (4.6%)	2 (2.0%)	1 (1.3%)
Dietitian	1 (0.8%)	1 (1.0%)	0
Exercise physiologist	1 (0.8%)	1 (1.0%)	0
Geriatrician	3 (2.3%)	2 (2.0%)	2 (2.6%)
Nurse—acute care services	2 (1.5%)	1 (1.0%)	0
Nurse—rehabilitation	27 (20.6%)	22 (21.8%)	19 (24.4%)
Physiotherapist	28 (21.4%)	23 (22.8%)	20 (25.6%)
Psychologist	2 (1.5%)	1 (1.0%)	1(1.3%)
Rehab Medicine physician	32 (24.4%)	27 (26.7%)	20 (25.6%)
Occupational therapist	19 (14.5%)	16 (15.8%)	11 (14.1%)
Social worker	5 (3.8%)	2 (2.0%)	1 (1.3%)
Speech therapist	1 (0.8%)	0	0
Other^a^	4 (3.1%)	3 (3.0%)	3 (3.8%)
Total	131 (100%)	101 (100%)	78 (100%)

^a^Researcher (*n* = 3); other therapist (*n* = 1, DR1 only)

**Fig. 2:**
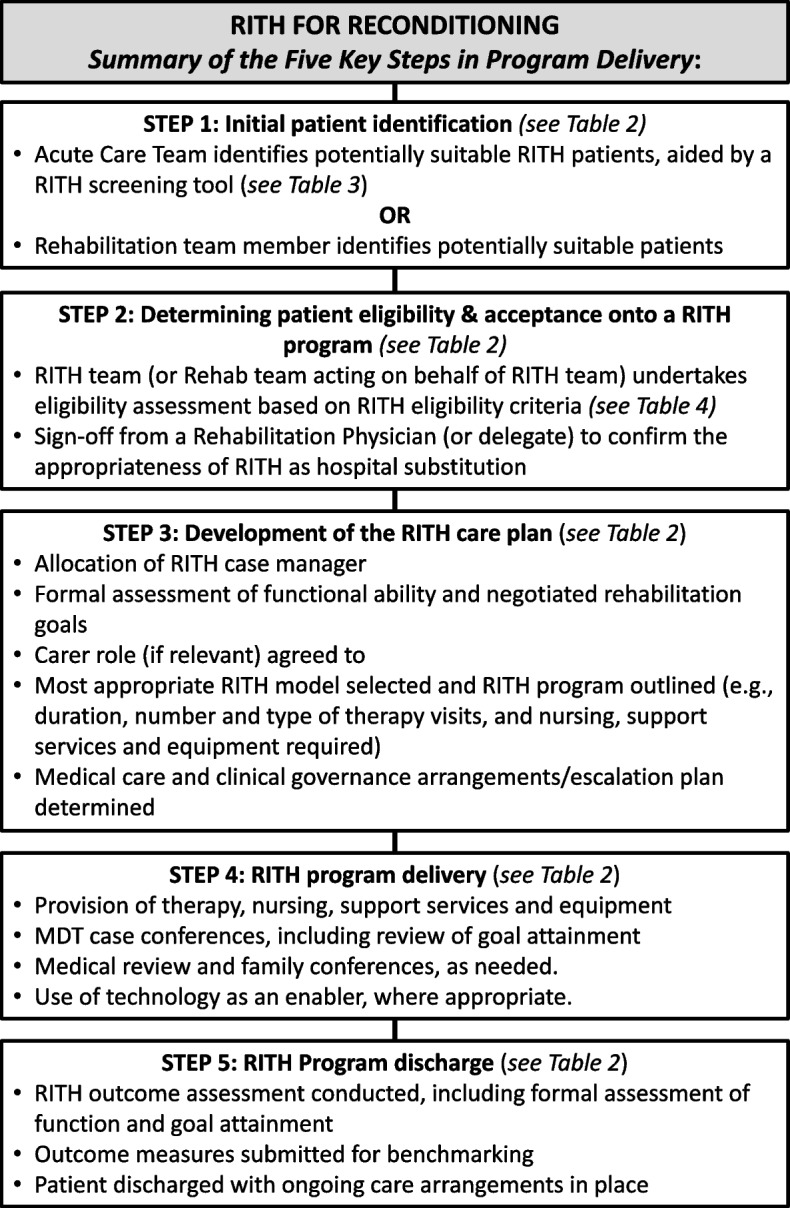
Key steps in RITH program delivery

Consensus on survey items led to the development of a model for RITH, comprising five key steps and the actions within each, which are summarised and illustrated in Fig. 2.

Consensus statements are reported in the text under these five key steps, and reference a corresponding Table and specific item number within that Table. The exact statement wording and level of support of items achieving consensus (shown along with the Delphi round in which consensus was achieved) are reported in Table 2 (i.e., T2-Item No.), Table 3 (i.e., T3-Item No.) or Table 4 (i.e., T4-Item No.). Table 3 lists consensus statements specifically relating to patient screening by an acute care team; Table 4 lists consensus statements specifically relating to patient eligibility; with Table 2 listing the remaining consensus statements. Items not achieving consensus are reported in Additional File 1 (i.e., A1-Item No.). Some items in A1 which did not achieve consensus initially, were modified in a following round, and subsequently achieved consensus. Survey items where multiple choice or item ranking were used are reported in Additional File 2 (i.e., A2-Item No.). Relevant participant comments from free text boxes are shown in the text in *italics*, with the corresponding Delphi round respondent number (i.e., DR-Respondent No.).

**Table 2:** Survey items reaching consensus^a^

Item no	Survey Item	%	Delphi Round
**STEP 1 Initial patient identification**			
S1.1	Members of a rehabilitation team should be the ones who identify patients who might be suitable for RITH	85.5	1
S1.2	Members of acute care teams are able to identify patients who might be suitable for RITH if they use a formal screening tool	79.4	1
S1.3	To be identified as potentially suitable for RITH, the patient’s carer (if there is a carer) must agree that RITH is a possible option	90.1	1
**STEP 2 Determining patient eligibility**			
S2.1	Members of a rehabilitation team should be the ones who determine a patient’s eligibility for RITH	84.6	1
S2.2	Members of acute care teams are able to determine a patient’s eligibility for RITH if they have used an appropriate assessment tool	73.8	1
S2.3	The RITH team should have the final say on each patient’s eligibility for RITH	92.3	1
S2.4	A rehabilitation physician (or their delegate) should sign off on each patient’s eligibility for RITH	77.7	1
S2.5	Where a home visit is **not** undertaken in determining patient eligibility for RITH, some alternate means of assessing the safety and suitability of the home environment should be undertaken (such as a checklist)	95.0	2
S2.6	In the absence of a home visit, a satisfactory initial assessment of the patient’s home environment could be done with a ‘virtual tour’ of the home	82.2	2
S2.7	Patients and carers considering RITH should also be informed about the option of inpatient rehabilitation (including the pros and cons of each) so they can make an informed decision about participating in RITH	91.1	2
S2.8	As part of the eligibility assessment for RITH, the RITH team and patient should jointly establish and agree on minimum achievable goals expected from a RITH program	98.7	3
S2.9	A written agreement for carers, which makes explicit the expectations and roles of a carer when a patient enters a RITH program, is desirable	81.2	2
S2.10	To be eligible for RITH, the patient’s carer (if there is a carer) must agree that the support available through RITH is sufficient for the carer to support the patient at home during their RITH program	95.4	1
S2.11	When required, paid support services (e.g. personal care, home help, meal services) should be available to patients on RITH programs, irrespective of whether they have a carer or not	94.1	2
S2.12	To be eligible for RITH, the patient must have a general practitioner who is willing to continue ongoing general medical care during their RITH program	71.5	1
S2.13	The acute care team is responsible for organising patient discharge from acute care, even though a patient might have been accepted on to a RITH program	84.4	1
S2.14	Discharge from acute care should not occur without liaising with a patient’s carer (if there is a carer)	96.1	1
S2.15	A RITH team member should work in collaboration with the acute care team to facilitate the patient’s discharge from acute care	90.6	1
S2.16	Acute care hospitals should not discharge medically unstable patients to RITH programs	93.7	1
S2.17	RITH programs should not accept medically unstable patients	89.1	1
**STEP 3 Development of the RITH care plan**			
S3.1	An initial RITH care plan that is done before the patient is discharged from acute care can be developed by only one multidisciplinary team member, as long as that person is experienced and able to take an interdisciplinary approach	78.2	2
S3.2	The patient cannot be properly assessed, and their RITH care plan cannot be properly developed, without a brief admission to a rehabilitation ward	73.1^b^	1
S3.3	A rehabilitation physician (or their delegate) should assess the patient before the initial RITH care plan is developed	78.5	1
S3.4	The patient’s RITH care plan should include an indicative program duration	93.1	1
S3.5	The patient’s RITH care plan should include an indicative number and type of therapy interventions	94.6	1
S3.6	Patient assessment tools used in RITH programs should include those that are, or will be, supported by the Australasian Rehabilitation Outcomes Centre (AROC)	77.7	1
S3.7	Flexibility in RITH program intensity and duration (within an approximate budget) should be available depending on patient preference and clinical situation	95.0	2
S3.8	Patient need should be the primary determinant of the intensity and duration of a RITH program	89.9	1
S3.9	Both Model 1 and Model 2 should be available RITH models	78.9	3
S3.10	Under Model 2, in general, the maximum length of a RITH program should be considered as 10 weeks	80.3	3
S3.11	Intensive ‘single discipline’ reconditioning rehabilitation programs should be available as a RITH model if the patient would need to remain in hospital, or cannot be safely discharged from hospital, without the availability of such a program	77.6	3
S3.12	A RITH service provider is best placed to provide these intensive ‘single discipline’ reconditioning rehabilitation programs because of the expertise available within the RITH team to monitor patient progress with goals and take corrective action if necessary	78.9	3
S3.13	To increase awareness of RITH, RITH for reconditioning should be included as a recognised post-acute care pathway	93.1	2
S3.14	When a Rehabilitation Physician or Advanced Trainee assesses a patient in acute care, RITH should be included as one of a range of rehabilitation options to be considered	96.0	2
S3.15	Where a patient is to have an elective procedure following which they might require reconditioning rehabilitation, then a discussion about RITH as a viable rehabilitation option should be commenced with the patient prior to their hospital admission	88.1	2
S3.16-S3.24 What do you think are important components of a RITH case manager’s role?			
	S3.16—Liaison between the different treating team members	94.6	1
	S3.17—Liaison with the patient and their carer (if they have one) about their care plan	97.7	1
	S3.18—Liaison with the patient’s RITH rehabilitation physician	93.0	1
	S3.19—Establishing with the patient’s GP / GP Practice how the GP wants to receive communication about their patient’s participation in RITH	91.5	1
	S3.20—Ongoing liaison with the patient’s general practitioner	82.2	1
	S3.21—Ensuring the provision of community support services where necessary	91.5	1
	S3.22—Ensuring that the patient care record is maintained and up-to-date	80.6	1
	S3.23—Organising case and family conferences	96.1	1
	S3.24—Managing the patient’s discharge from the RITH program	82.9	1
S3.25	A case manager needs to be clinical	85.1	2
S3.26	A case manager should have administrative support	84.2	2
S3.27	Where available, the RITH Team (or an affiliated in-reach rehabilitation service) should commence rehabilitation with the patient still in acute care	74.0	2
S3.28	The rehabilitation medicine physician should have a central role in the provision of RITH, as they do in inpatient rehabilitation units	79.5	1
S3.29	The primary responsibility for rehabilitation care during RITH sits with the rehabilitation physician	72.7	1
S3.30	Given that a rehabilitation physician must be involved in a patient’s RITH program, the rehabilitation physician bears responsibility for the oversight of the patient’s RITH program	87.0	2
S3.31	The rehabilitation physician must be readily contactable by a RITH therapist or nurse (and vice versa) when either needs to discuss patient care	97.0	2
S3.32	As a principle of care, the rehabilitation physician and the patient’s general practitioner should collaborate on relevant patient care decisions	83.6	1
S3.33	The extent (frequency of review) of involvement of the rehabilitation physician will be determined by the complexity and needs of the patient	94.0	2
S3.34	Other appropriately skilled medical specialists (e.g., geriatricians for patients in the geriatric aged group) could fulfil a similar role to the rehabilitation medicine physician in RITH for reconditioning	72.4	3
S3.35	To be eligible for RITH, the patient must have a general practitioner who is willing to continue ongoing general medical care during their RITH program	71.5	1
S3.36	The role of the GP will depend on the level of general medical care that can be provided by the RITH program	81.8	2
S3.37	The patient must have a means of accessing their GP during their RITH program	87.6	1
S3.38	As the duration of the RITH program increases, the patient’s GP should have more responsibility for providing general medical care	80.8	2
S3.39	A full discharge summary should be provided to the GP when the patient leaves acute care	100.0	1
S3.40	The GP needs to be informed when the patient has been discharged home to commence RITH	99.2	1
S3.41	Information describing the general responsibilities of RITH team members should be provided to GPs at the start of their patient’s RITH program	92.9	2
S3.42	Communication supplied to a patient’s GP should include the patient’s RITH care plan and the patient’s anticipated goals from RITH	92.2	1
S3.43	An MBS-approved GP case conference with members of the RITH team towards the end of the RITH program is ideal	70.5	1
S3.44	Each RITH service must have an overarching clinical governance framework under which RITH programs are delivered	98.7	3
S3.45	In all RITH models, it is important to ensure that clinical governance arrangements (such as responsibility for medical care and emergency management plan) are determined and known to everyone involved (RITH team, GP and patient/carer) at the outset of the RITH program	98.7	3
S3.46	There must be a reporting system in place for all adverse events and near miss incidents for patients participating in RITH programs	99.2	1
S3.47	The Australasian Faculty of Rehabilitation Medicine (AFRM) should be responsible for developing specific standards for RITH programs	71.9	1
S3.48	AFRM standards should be used to guide and optimise the care of patients in a RITH program	72.7	1
**STEP 4 RITH program delivery**			
S4.1	As long as team members know and understand their professional boundaries, an interdisciplinary approach can be an appropriate model of service provision for RITH for reconditioning	88.5	3
S4.2	A suitably skilled nurse/s should be part of a RITH team	91.1	2
S4.3	Allied health assistants have an important role to play in RITH	93.1	2
S4.4	Rehabilitation in the home (RITH) patients should receive as comprehensive a rehabilitation service as they would have received if they had been undergoing inpatient rehabilitation	87.1	2
S4.5	Reconditioning following cancer should include psychosocial care delivered by a social worker and/or a psychologist	89.0	2
S4.6	Multi-disciplinary team case conferences should feature in each patient’s RITH program	94.5	1
S4.7	Family conferences (if relevant) should feature in each patient’s RITH program	89.1	1
S4.8	Resources (e.g. printed or electronic) that describe exercises or other rehabilitation therapies should routinely be provided to help patients (and carers) do their therapy when the therapist is not with them	95.0	2
S4.9	If the carer is to partner in the patient’s rehabilitation (e.g. supporting therapy without a therapist present), then the RITH program must include time for carer education	98.0	2
S4.10	Providing therapy at community locations (for example, a local community centre or gym) could be considered to fit within a RITH program	75.2	1
S4.11	Providing therapy at health facilities (e.g., hydrotherapy pool, hospital gym or clinical space) could be considered to fit within a RITH program (as long as it did not involve the ‘admission’ of the patient to that hospital and did not breach other funding rules)	79.1	1
S4.12	A RITH service could use an external brokerage model to provide personal care, home help and meals when required by patients while they undergo RITH	85.1	2
S4.13	There is a role for technology (telehealth/telerehabilitation) within RITH programs	93.0	1
S4.14	Technology can be an effective means of providing rehabilitation therapy in a patient’s home during a RITH program	82.9	1
S4.15	Technology can be an effective means for a rehabilitation physician to monitor a patient’s progress during RITH	93.0	1
S4.16	When rehabilitation physicians utilise telehealth/telerehabilitation, its use should be guided by the clinical situation, the availability of technology and the ability of the patient to participate in telehealth/telerehabilitation	96.2	3
S4.17	Where IT literacy, or sensory or cognitive deficits limit the ability of a patient and/or carer to use the technology, a RITH MDT member should be in the patient’s home to assist during any telehealth/telerehabilitation session with a rehabilitation physician	91.0	2
S4.18	It is acceptable clinical practice during a video consultation with the rehabilitation physician that a member of the RITH MDT be in the patient’s home to facilitate demonstration of the patient’s functional ability, where the patient cannot otherwise reliably do so	89.0	2
**Step 5 RITH Program Discharge**			
S5.1	RITH patient outcome data should be based on functional measures (e.g., FIM change)	84.5	1
S5.2	RITH patient outcome data should be based on the degree of achievement of negotiated patient goals	96.9	1
S5.3	PREMs (Patient Reported Experience Measures) and PROMs (Patient Reported Outcome Measures) are contemporary measures that should be included in RITH outcome assessment	89.5	3
S5.4	RITH data should be submitted to AROC for benchmarking	85.3	1
S5.5	Patient outcome tools used in RITH programs should include those that are, or will be, supported by the Australasian Rehabilitation Outcomes Centre (AROC)	82.9	1
S5.6	Ideally, for the purposes of benchmarking for RITH, there should be a mandatory set of AROC-supported assessment/outcome tools used by all RITH services	92.1	3
S5.7	Admission to inpatient rehabilitation should be available to RITH patients where progress has failed, and inpatient rehabilitation may assist	92.1	2
S5.8	An acceptable key performance indicator (KPI) for subsequent admission to inpatient rehabilitation following a ‘failed’ RITH for reconditioning program is ≤ 10%	81.8	3
S5.9	In a well-functioning RITH program, acute hospital readmission rates should be as low as or lower than acute hospital readmission rates following inpatient rehabilitation	85.0	2
S5.10	Discharge from the RITH program should occur as soon as the patient’s goals have been achieved	85.3	1
**Budgetary Factors**			
BF.1	The available budget for a patient’s RITH program is more important than the patient’s clinical care needs	87.4^b^	1
BF.2	The cost of a patient’s individual RITH program should be no more than the cost of a comparable inpatient rehabilitation episode	72.4	1
BF.3	Any RITH program/model should have an endpoint for service delivery	93.4	3
BF.4	There should be some budget flexibility between individual patient RITH programs, as long as the overall RITH service is able to work to its budget for a given level of activity	95.0	2
BF.5	Expenditure beyond a baseline budget amount for each patient’s RITH program should sit with someone above the case manager/team delivering care	75.0	2
BF.6	There should be processes put in place to allow for the activation of a predetermined extension of a RITH program if it is likely that further quantifiable improvements in function and quality of life can be evidenced (but limits would still apply)	92.0	2
BF.7	It is unlikely that any funder (public or private) will agree to support RITH for reconditioning unless budget parameters have been set and agreed to for the RITH program	92.0	2
BF.8	Any RITH service, whether publicly or privately funded, must work to a budget (either an individual budget per patient OR an overall budget for the RITH service)	96.1	3
BF.9	Predictive models used to develop a costing model for RITH are helpful at an overall program level but should not be strictly applied at the individual patient level	85.0	2
BF.10	Prior to implementing a RITH for reconditioning service, the key ‘decision makers’ (i.e., the rehabilitation physician and RITH case managers) should receive formal education on setting, monitoring and managing individual RITH program budgets	89.0	2
BF.11	A schedule of costs (that is, a pricing schedule or pricing tool) for each type and mode of delivery of therapy interventions could be used to aid in the development and costing of the patient’s RITH care plan	89.0	2
BF.12	It will be attractive for health service administrators in the public system if the availability of RITH for reconditioning allows more patients to receive rehabilitation for the same financial outlay	91.0	2
BF.13	It will be attractive for private health insurers if the same patient outcomes can be achieved by RITH for less cost than comparable inpatient rehabilitation	95.0	2
BF.14	The nature of how a budget for RITH is applied may need to vary between public and privately funded RITH models	72.4	3
**Potential utilisation of RITH**			
PU.1	RITH for reconditioning for patients following medical illness, surgery, or treatment for cancer should be widely available for appropriate patients	96.1	1
PU.2	It will be too complex to design a model of care for RITH for reconditioning	85.8^b^	1
**Miscellaneous**			
M.1	Patients admitted to an inpatient rehabilitation ward usually require treatment by more than one allied health discipline	92.1	1

^a^Consensus achieved where ≥ 70% of participants agree or disagree^b ^with statement. Table shows survey item, the percentage of participants agreeing or disagreeing^b ^with statement, and the Delphi round in which survey item appeared

^b^ Indicates percent of participants disagreeing with item

**Table 3:** Acute care team patient screening tool. Consensus Delphi items

Item No	Survey items	Percent agreement	Delphi round
1	The patient has become deconditioned following medical illness, surgery or a cancer diagnosis or treatment	94.8	3
2	The patient is medically stable, or approaching medical stability, but has ongoing functional deficits that could be supported by a RITH program	97.4	3
3	The patient was living at home prior to admission to hospital, and the intention is for them to return home	88.5	3
4	The patient might ordinarily be considered for transfer to inpatient rehabilitation	79.5	3
5	The patient is willing to consider doing their rehabilitation at home	96.2	3
6	The patient is considered likely to be an active participant in their rehabilitation	89.7	3
7	If there is a carer, the carer is willing to consider the patient doing their rehabilitation at home	94.9	3

**Table 4:** Patient eligibility criteria. Consensus Delphi items

Item No	Survey items	Percent agreement	Delphi round
1	The patient was living at home prior to admission to hospital, and the intention is for them to return home	93.1	2
2	The patient will be functionally independent inside their home in terms of mobility (with or without aids) or has a willing and able carer who can supervise/assist with mobility	95.0	2
3	The patient will be functionally independent at home with toileting and personal care (with or without aids) or has a willing and able carer who can supervise/assist with toileting and personal care	93.1	2
4	The patient can manage the basic necessities of living (such as meal preparation or obtaining meals, or doing their laundry) or has a willing and able carer who can assist with these tasks, or these tasks can be provided under the RITH program	91.1	2
5	The home environment is considered to be safe for the patient whilst they undergo a RITH program	97.0	2
6	The home is accessible for the patient (patient can enter and exit the home)	86.1	2
7	The patient is deemed medically stable for discharge home into a RITH program	97.0	2
8	The patient has sufficient cognition to participate in a RITH program, or has a willing and able carer who can assist the patient to participate actively	90.1	2
9	The patient is agreeable to having rehabilitation in the home	99.0	2
10	The patient is motivated to participate in rehabilitation	96.0	2
11	The home environment does not pose a safety risk to staff	98.0	2
12^a^	The patient’s falls risk is manageable^b^ and/or patient autonomy concerning falls risk has been considered^c^	> 70.0	3
13^a^	Minimal achievable goals for RITH can be agreed upon^d,e^	> 70.0	2, 3

^a^Derived from more than one Delphi item

^b^Survey item in DR3 read:’ A falls risk assessment should be undertaken by the RITH/Rehabilitation team in their assessment of patient eligibility for RITH, and only patients deemed to have a manageable falls risk should be accepted onto a RITH program’ (71.8% agreement)

^c^Survey item in DR3 read: ‘Patient autonomy should be taken into account when considering whether patients deemed to be of high falls risk should be accepted onto a RITH program’ (78.2% agreement)

^d^Survey item in DR2 read: ‘Patients selected for RITH should be those for whom their desired outcomes are likely to be achievable within the available program intensity and duration’ (90.1% agreement)

^e^Survey item in DR3 read: ‘If minimum achievable goals for RITH cannot be agreed upon, then the patient should not be offered RITH but should be offered an alternate care pathway, that might include inpatient rehabilitation’ (81.6% agreement)

### Step 1: Initial patient identification

There was consensus for members of a rehabilitation team being the ones to initially identify patients potentially suitable for RITH (See T2-S1.1). However, it was also agreed that acute care teams, aided by the use of a formal screening tool, could identify potential patients (T2-S1.2). Table 3 lists supported items for use in screening potential patients by acute care teams.

### Step 2: Determining patient eligibility and acceptance onto a RITH program

Following the identification of potentially suitable patients, eligibility for RITH should be determined by the RITH team or a rehabilitation team acting on behalf of the RITH team (T2-S2.1). The RITH team should have the final say on eligibility (T2-S2.3), with sign-off by a rehabilitation physician (or their delegate) (T2-S2.4) to confirm the appropriateness of RITH as hospital substitution. Table 4 lists supported items for determining patient eligibility and includes items relating to patient function, cognition, motivation, available support, and the home environment. Assessment of the safety and suitability of the home was considered essential, with alternatives to a physical home visit (e.g., checklist or virtual tour) being acceptable (T2-S2.5, S2.6). It was deemed important in considering eligibility that only patients whose goals are likely to be achievable within a RITH program should be selected (T4-13). Consensus was not reached on the patient’s degree of frailty (as scored by the Clinical Frailty Scale [23]) being a determinant for eligibility (A1-6).

‘High falls risk’ in the patient as an exclusion criterion for RITH fell just short of consensus (69.3%, A1-7), with comments in DR2 suggesting that high falls risk is common in rehabilitation patients. Consensus on eligibility was subsequently achieved in DR3 for a patient having a ‘*manageable*’ (DR3-43) falls risk, alongside consideration of ‘*patient autonomy’* (DR3-26) around this risk. Comments supported ‘*falls risk mitigation strategies*’ (DR3-11) such as ‘*falls education*’ (DR3-51), ‘*the provision of equipment and aids…[and] carer supervision during mobility tasks*’ (DR3-11); with eligibility decisions informed by patient and family ‘*insight*’ (DR3-63) and whether they are likely to be ‘*compliant*’(DR3-59) with falls mitigation strategies.

Patients and their carers should have a clear understanding of the available rehabilitation options (pros and cons of RITH versus inpatient rehabilitation) (T2-S2.7), and jointly established minimum achievable goals must be agreed prior to admission to RITH (T2-S2.8). A written agreement for carers, making explicit the expectations and roles of a carer during RITH, is desirable (T2-S2.9). Carers must agree that the support available through RITH is sufficient for them to support the patient at home (T2-S2.10). Further, paid support services should be available to patients on RITH programs when needed (e.g., personal care, home help, meal services) (T2-S2.11).

It was agreed that the acute team remains responsible for discharge from acute care (T2-S2.13). The RITH team should work with them to facilitate patient discharge (T2-S2.15) and ensure that liaison with carers occurs (T2-S2.14). Acute hospitals should not discharge medically unstable patients to RITH (T2-S2.16), and medically unstable patients should not be accepted onto RITH programs (T2-S2.17).

### Step 3: Development of RITH care plan

DR1 sought the best time for the development of the RITH care plan. Comments viewed the care plan as an ‘*evolving process*’ (DR1-111) with an ‘*initial basic plan*’ (DR1-88) in place before the patient leaves acute care ‘*reflecting the person’s status on discharge*’ (DR1-99). A more comprehensive plan would follow the multidisciplinary team (MDT) case conference, being modified when the patient’s ‘*goals become clearer once they are at home*’ (DR1-52). Clarity and consensus on who can develop the initial care plan was confirmed in DR2 (T2-S3.1). A brief admission to a rehabilitation ward for assessment and RITH care plan development is not needed (T2-S3.2).

A rehabilitation physician or their delegate should assess the patient before the initial care plan is developed (T2-S3.3). The care plan should include an indicative program duration, and number and type of therapy sessions (T2-S3.4, S3.5). There was consensus for using assessment tools that are, or would be, supported by AROC (T2-S3.6).

#### RITH service delivery model

Participants were initially presented with two different models of RITH (DR2), with the assumption that the resources required would be similar in both: Model 1 (RITH mirrors the equivalent inpatient rehabilitation episode in terms of duration and intensity); Model 2 (RITH is longer than the equivalent inpatient episode, but of less intensity). Model 1 was favoured by 36.4% of participants, Model 2 by 47.5%, with 16.2% undecided (A2-4); but flexibility in RITH program intensity and duration (within an approximate budget) was supported (T2-S3.7), determined by patient need (T2-S3.8). Consensus was achieved in DR3 for there being two models of RITH available (T2-S3.9), that is, Model 1 (as described above) and Model 2 (with the descriptor for ‘intensity’ revised from ‘less’ to ‘less or varied’). The maximum duration of Model 2 programs was considered to be around ten weeks (T2-S3.10).

While participants in DR2 agreed that patients admitted to an inpatient rehabilitation ward usually required treatment by more than one allied health discipline (T2-M.1), opinions were divided on whether this was required for ‘RITH as hospital substitution’ (A1-13, 39.6% agreement; 45.5% disagreement). Comments suggested that ‘*even if the patient needs only one active allied health service, the patient may still benefit from case conference *etc. *of RITH*’ (DR2-7) and that ‘*sometimes patients have quite severe single system issues [which] would [otherwise] need a stay in hospital*’ (DR2-30). Reworked statements achieved consensus in DR3 for a third RITH model—intensive ‘single discipline’ programs where the patient would otherwise need to remain in hospital (T2-S3.11), overseen by the RITH team because of their expertise, and with access to other RITH MDT resources if necessary (T2-S3.12).

To increase awareness of RITH for reconditioning, participants agreed that RITH should be included as a recognised post-acute pathway, considered as a rehabilitation option when a patient is assessed for rehabilitation in acute care, and where relevant, discussed with patients prior to a planned hospital admission (T2-S3.13- S3.15).

#### Case manager

In DR1 it was suggested to participants that a case manager may be important to the success of a RITH program. Consensus was achieved on suggested components of a case manager’s role (see T2-S3.16-S3.24). The case manager should be clinical (T2-S3.25); though not necessarily a rehabilitation nurse (A1-27), with comments indicating other disciplines would also be suitable. Choosing from a list provided (DR1), the preferred case manager for 60.3% of participants was a member of the RITH team treating the patient, and for another 30.2% of participants it was a team member who was not treating the patient. Few participants (6.4%) felt no case manager was needed (See A2-2). Having administrative support for a case manager was supported (T2-S3.26).

#### Medical care and clinical governance during RITH

It was agreed that the rehabilitation physician has a central role and primary responsibility for rehabilitation care during RITH (T2-S3.28 – S3.29), is responsible for overseeing the patient’s program (T2-S3.30), should be readily contactable by the MDT to discuss patient care (T2-S3.31), and should collaborate where relevant with the patient’s general practitioner (T2-S3.32). The frequency of review by the rehabilitation physician will depend on the complexity and needs of each patient (T2-S3.33). Other appropriately skilled medical specialists (e.g., geriatricians for patients in the geriatric age group) could fulfil a similar role to the rehabilitation medicine physician in RITH for reconditioning (T2-S3.34).

Consensus on the primary responsibility for management of the ongoing general medical care of the RITH patient during their program (A1-18, 22, 23) could not however be reached without clarification on system issues. These issues included the level of general medical care available through the RITH program (T2-S3.36) and funding rule conflicts between an ‘admitted’ versus ‘non-admitted’ patient (with those receiving ‘RITH as hospital substitution’ being regarded as ‘admitted patients’ in Australia). One participant noted, ‘*this entails the patient being both an inpatient ([admitted]) … and an outpatient ([non-admitted]) … simultaneously*’ (DR1-106) Others raised questions about the ‘*access and the availability of GPs to provide the necessary support’ (DR1-127)* and that *‘not everyone has a GP*’ (DR1-98). However, participants supported patients having a means of accessing their GP during their RITH program, and that GPs should assume more responsibility for providing general medical care as RITH program duration increases (T2-S3.37, S3.38). Communication with GPs is key and should include acute hospital discharge summaries, information about the responsibilities of the RITH team, their patient’s care plan and goals, and case conferences that include the GP (T2-S3.39-S3.43).

An overarching clinical governance framework which is widely understood (T2-S3.44-S3.45), and an adverse event and near miss incident reporting system (T2-S3.46) are required. There was support for specific standards for RITH programs as hospital substitution being developed (T2-S3.47-S3.48).

### Step 4: RITH program delivery

#### Teamwork

In DR2 we defined multidisciplinary teamwork as ‘therapists working in an integrated manner, but in parallel with each other’, and interdisciplinary teamwork as ‘therapists working in an integrated manner and striving to do cross disciplinary work wherever possible (but still within their scope of practice)’. An interdisciplinary model was preferred (57%), followed by a multidisciplinary model (37%), with 6% of participants unsure (A2.3). With further qualification, consensus was achieved (DR3) for an interdisciplinary approach so long as team members knew and understood their professional boundaries (T2-S4.1). Comments suggested that therapists ‘*provide specialist care within [their] own profession*’ (DR2-71) but there was also a recognition of ‘*substantial cross over between allied health roles*’ (DR2-9) which may increase with experience. It was felt that ‘*highly experienced clinicians [are needed for RITH]. These clinicians are likely to be able to function in an interdisciplinary team*’ (DR2-27). Participants described interdisciplinary teamwork as having ‘*greater flexibility*’ (DR2-1), being a ‘*more efficient service for patients*’ (DR2-29), and necessary ‘*because of resources availability*’ (DR2-7). In summary, there was a sense that the ‘*team should work as collaboratively as possible to achieve [patient] goals*’ (DR2-17). RITH teams should include suitably skilled nurses (T2-S4.2) and allied health assistants (T2-S4.3).

#### Other aspects of care provision

RITH patients should receive as comprehensive a service as they would have received if undergoing inpatient rehabilitation (T2-S4.4). DR1 comments suggesting the need for psychosocial support following cancer were tested in DR2 and supported (T2-S4.5), but comments indicated it may also be needed more broadly. RITH programs should include case and family conferences (T2-S4.6, S4.7), the routine provision of resources (e.g., printed/electronic) to help patients (and carers) with therapy in the therapist’s absence (T2-S4.8), and carer education to enable carers to partner in the patient’s rehabilitation (T2-S4.9). RITH programs can include therapy at locations other than the patient’s home (e.g., local gym or hydrotherapy pool at a health facility) providing funding rules (of government or private health insurance) are not breached (T2-S4.10 or S4.11). An external brokerage model as a means of supplying necessary support services, such as home help, was supported (T2-S4.12) if not directly available through the RITH service.

#### Technology

Consensus on a role for telehealth/telerehabilitation in providing and monitoring therapy was achieved (T2-S4.14 – S4.16). Comments indicated that some patients struggle with technology, and its use ‘*should not replace face-to-face entirely*’ (DR1-121). To assist patients during telehealth/telerehabilitation sessions with the rehabilitation physician, a MDT member could be present in the home when necessary (T2-S4.17, S4.18).

### Step 5: RITH Program Discharge

Outcome data should include functional measures, degree of achievement of negotiated patient goals, patient reported experience measures (PREMs) and patient reported outcome measures (PROMs) (T2-S5.1–S5.3). Comments raised concerns about the suitability of the FIM ‘*as [RITH patients] are usually more independent than inpatient[s]*’ (DR1-114). Outcome data should be submitted to AROC for benchmarking (T2-S5.4-S5.5), with consensus achieved for a mandatory set of AROC-supported assessment and outcome tools (S5.6). Comments indicated these needed to be ‘*meaningful*’ (DR3-2) with ‘*input from different stakeholders*’ (DR3-51) and allow for ‘*comparison…between RITH and inpatient rehabilitation*’ (DR3-25).

Fall-back admission to inpatient rehabilitation should be available where patients fail to progress on RITH (S5.7), with a key performance indicator of ≤ 10% for subsequent admission to inpatient rehabilitation considered acceptable (T2-S5.8). Also, in a well-functioning RITH program, acute hospital readmission rates should be no higher than occurs following inpatient rehabilitation (T2-S5.9). Discharge from RITH should occur as soon as a patient achieves their goals (T2-S5.10).

### Budgetary Factors

The patient’s clinical care needs were seen as more important than the available budget for their RITH program (T2-BF.1), but participants also agreed that RITH costs should be no more than comparable inpatient episodes (T2-BF.2) and that RITH programs/models need an endpoint for service delivery (T2-BF.3). Budget flexibility between individual patient programs was supported if the overall RITH service was able to work to its budget (T2-BF.4). Appropriate processes for approval of additional expenditure and RITH program extension are needed (T2-BF.5-BF.6).

Budget parameters are necessary for funders (T2-BF.7). Any RITH service (publicly or privately funded) must work to a budget (either an individual budget per patient or an overall budget for the RITH service) (T2-BF.8). Predictive models used to develop a costing model for RITH can be helpful at an overall program level but should not be strictly applied at the individual patient level (T2-BF.9). Key decision makers should receive formal education on setting, monitoring and managing individual program budgets, and on using a schedule of costs or pricing tool to aid in the development and costing of patient RITH care plans (T2-BF.10-BF.11). RITH could be an attractive option in both the public and private systems (T2-BF.12-BF.13).

### Potential utilisation of RITH

There was strong support for RITH for reconditioning being widely available for appropriate patients (T2-PU.1). Participants did not believe that the design of a model of care for RITH would be too complex (T2-PU.2). Anticipated utilisation of RITH was tested in DR3 by asking participants to estimate its likely utilisation for each of the six reconditioning case-mix classes used in Australia (AN-SNAP classes 4AR1 through 4AR6 [1]). These classes are explained with Table 5, along with participant responses. Twenty-one participants (of the 78 who participated in DR3) who felt they were sufficiently experienced with AN-SNAP to respond to the questions estimated that around two thirds of patients in the first two classes (4AR1 and 4AR2, which account for over one half of all inpatient reconditioning episodes in Australia [24]) would likely be suitable for RITH. They also estimated that around three-quarters of these suitable patients might want to take part in RITH if it were available (Table 5).

**Table 5:** Participant estimates of RITH utilisation by AN-SNAP class^a^

AN-SNAP (Version 4) class^b^	What percentage of reconditioning patients in the following AN-SNAP classes do you think are likely to be suitable for RITH?		What percentage of suitable reconditioning patients would you expect to actually want to take part in a RITH program if one were available?	
	Median %	IQR	Median %	IQR
4AR1	74.0	39.0	80.0	30.0
4AR2	65.0	25.0	74.0	35.5
4AR3	41.0	31.5	53.0	31.5
4AR4	50.0	45.0	54.0	41.5
4AR5	30.0	42.0	36.0	47.0
4AR6	11.0	36.0	20.0	47.0

^a^Participant estimates of percentage of patients likely to be suitable for RITH by AN-SNAP class, and participant estimates of the percentage of likely suitable patients who may want to take part in RITH. Median scores and interquartile range (IQR), *n* = 21 participants

^b^The Australian National Subacute and Non-Acute Patient classification (AN-SNAP) is used to determine activity-based funding for admitted subacute care services in public hospitals. The general code 4AR refers to patients requiring reconditioning. Patients are assigned AN-SNAP reconditioning classes on admission to rehabilitation programs. These classes are based on different levels of patient function as shown below (24):

4AR1 Reconditioning, weighted FIM motor 67‐91

4AR2 Reconditioning, weighted FIM motor 50‐66, FIM cognition 26‐35

4AR3 Reconditioning, weighted FIM motor 50‐66, FIM cognition 5‐ 25

4AR4 Reconditioning, weighted FIM motor 34‐49, FIM cognition 31‐35

4AR5 Reconditioning, weighted FIM motor 34‐49, FIM cognition 5‐ 30

4AR6 Reconditioning, weighted FIM motor 19‐33

## Discussion

Numerous studies refer to home-based rehabilitation (see for example, [25–28]) but there is little research addressing rehabilitation at home for patients with ‘generalised deconditioning not attributable to any of the other impairment groups’ (that is, AROC impairment codes 16.1, 16.2 and 16.3 [1]). We believe this is the first study to seek to establish consensus on a model for RITH as hospital substitution for these patients. We used the Delphi technique, a popular consensus group method in health care research [29], to draw upon the knowledge and expertise of professionals from a range of disciplines and geographic jurisdictions to achieve agreement on over 130 statements. These related to the RITH patient journey, clinical governance, and budgetary considerations (Table 2); and included items for initial patient screening (Table 3), patient eligibility (Table 4) and case manager roles (Table 2). Consensus on survey items led to the development of a model for RITH, comprising five key steps and the actions within each (Fig. 2).

It is important to highlight for an international audience that inpatient rehabilitation is a recognised category of subacute clinical care within the Australian hospital system. Rehabilitation medicine physicians, or similarly qualified and credentialed physicians assume overall patient responsibility for inpatient episodes (that is, ‘the name over the bed’). In keeping with this, participant consensus on RITH as hospital substitution for reconditioning reported in this study aligns strongly with the criteria that define an episode of ‘rehabilitation care’ given by the Australian Government’s Independent Hospital Pricing Authority (IHPA) [30] specifically:

1. The primary clinical purpose or treatment goal is improvement in the functioning of the patient (e.g., T2-S2.8, S3.8, S3.11, S5.2, S5.7, S5.8, S3.42, BF.6; T3-2; T4-13)
2. The patient is capable of active participation (e.g., T3-6; T4-8, T4-10)
3. The delivery of rehabilitation care is under the management of, or is informed by, a clinician with specialised expertise in rehabilitation (e.g., T2-S3.28, S3.29, S3.30, S3.31, S3.33, S3.34)
4. There is an individualised, documented, multi-disciplinary management plan (e.g., T2-S3.1, BF.11)) which comprises negotiated goals (T2-S2.8, S3.42; T4-13) within stated time frames (T2-S3.4) and formal assessment of functional ability (T2-S5.1, S5.2, S5.3).

### Patient eligibility factors

Considerable variation in views amongst inpatient staff about patient suitability for rehabilitation [31], and for early supported discharge programs [32] has been reported in the literature, which impacts equity of access for patients and makes the adoption of agreed service models problematic. Our findings support a two-step process (i.e., screening, followed by a more detailed eligibility assessment), with agreement being reached on relevant items for both (Tables 3 and 4). This should assist in addressing referral inconsistency and in improving the efficiency of patient referrals to RITH.

Eligibility items include a range of factors which are generally not considered in determining eligibility for inpatient rehabilitation. For example, whether the home environment is safe for both patient (while undergoing their RITH program) and staff (no safety risk posed); and whether the patient will be able to manage the necessities of living (such as meal preparation, doing laundry), or if not, that these tasks can be provided for under the RITH program (e.g., using external brokerage if required). The willing assistance of the patient’s carer (where there is one) was also seen as key to eligibility, and participants supported written documentation for carers to consider and agree to, which makes explicit the expectations and roles of a carer when a patient enters RITH. This would address what Dow and McDonald [33] identified, that carers are usually consigned a marginal status within home-based rehabilitation programs and feel bound by an “invisible contract” to carry out substantial care-work that would otherwise have been undertaken by hospital staff. Finally, while high falls risk is generally not an exclusion criterion for inpatient rehabilitation [34], our findings suggest that when balancing falls risk with program benefit in the RITH setting, the situation may be more nuanced. Survey participants recognised that patients in the reconditioning impairment category are likely to have a degree of falls risk and the Delphi process settled on that risk being a ‘manageable’ one, rather than there being a specific level of falls risk that would exclude someone from a program, with patient autonomy, insight, compliance, and mitigation strategies to be considered. Further, as frailty and falls risk are strongly related [35, 36] it is consistent that survey participants did not also agree on a level of frailty as an inclusion or exclusion criterion for acceptance onto RITH.

In response to participant comments about how practice has had to adapt to the COVID-19 pandemic, we tested and received confirmation of the idea that a “virtual” tour of the patient’s home to assess the home environment is feasible. Interestingly, a scoping review of the literature published prior to the pandemic identified that the use of technology within occupational therapy home assessments had been generally underutilised [37], but that its use appeared to be feasible and acceptable, with benefits in terms of rapid visual access of a property, enhanced resource utilisation (e.g. elimination of travel time) and potential cost-effectiveness [37, 38].

### Program factors

Participants supported three RITH service delivery models—intensive and short term (mirroring closely an inpatient episode, which for example averages 12.1 days and 15.2 days for AN-SNAP classes 4AR1 and 4AR2, respectively [24]); variable and longer term, generally to a maximum of ten weeks duration; and intensive single discipline where the patient cannot be discharged from hospital without such a program. These models suggest the possibility of better tailoring programs to client needs. For example, Model 2 may allow some patients (e.g., with frailty or comorbid cognitive impairment or dementia) to engage in a program of therapy to achieve their goals at a slower pace and with less fatigue [39, 40] than may be experienced with Model 1. Consensus was for all three models to be delivered by a RITH service provider because of the expertise within the RITH team to monitor progress and take corrective action when needed. Indeed, co-ordinated MDT input was found to provide better outcomes for stroke patients in a Cochrane Review of early discharge with rehabilitation at home [13].

While not supporting any specific discipline as being the ideal RITH case manager, participants were of the view that a suitably skilled nurse should be part of the RITH MDT. Six roles for the nurse within the multi-professional rehabilitation team have been described for the inpatient setting, as follows: ‘assessment, co-ordination and communication, technical and physical care, therapy integration and therapy carry-on, emotional support, and involving the family’ [41]. While similar rehabilitation nurse roles will likely exist in RITH, these roles will probably be shared across RITH team members where an interdisciplinary approach to care is adopted.

There was strong support by participants for a mandatory agreed set of assessment and outcomes tools to enable benchmarking of RITH services. Around 98% of all inpatient rehabilitation episodes in Australia are reported to AROC [42]. This enables a robust analysis of clinical information for the purposes of reporting and improving outcomes, with AROC publishing comparative data biannually for inpatients [42]. However, the collection of ambulatory data by AROC remains in its infancy in Australia, and is hindered by the diverse range of service models in operation and the lack of agreed outcome measures [14]. Further, the opportunity to compare outcomes from inpatient and ambulatory settings may be lost where tools appropriate for both settings cannot be identified. The impact of heterogeneity of measurement tools on comparative research is illustrated by a systematic review of inpatient versus home-based rehabilitation for older adults with musculoskeletal disorders, which found 21 different assessment tools across 12 studies, with the most frequent tools only reported in three studies [43].

The identification of tools for RITH is challenging, particularly so for the reconditioning impairment category which covers a diverse clinical group. More patient-centric outcome tools may be needed, such as PREMs and PROMs and goal attainment scales. These were all supported by survey participants. Goal attainment is likely to be especially important because RITH patients may experience ceiling effects on standard functional measures (e.g., FIM), and reaching goal attainment was an agreed point of readiness for discharge from RITH.

### Clinical Governance and budgetary factors

A clear and widely understood system of clinical governance that ensures the continuing delivery of safe and high-quality care as the patient transitions from the hospital environment to the community was strongly supported. This included consensus on specific roles for the acute care team, the rehabilitation physician, the RITH team, and the case manager. Specific standards for RITH to be developed by the Australasian Faculty of Rehabilitation Medicine were supported. These could be similar to those that already exist for inpatient rehabilitation [44]. A clear medical escalation plan is also needed. Actions to integrate primary care (such that the patient’s GP can provide ongoing general medical care to the patient at home) into the RITH as hospital substitution program were supported but challenged by existing funding models. While there has been little research in Australia on vertical integration (e.g., between state-funded public hospitals and largely federally-funded practice in the community), significant funding model barriers to more integrated care models have been noted [45].

## Strengths and limitations

This was a large Delphi survey which was successful in retaining participants from a range of disciplines over three rounds, to achieve consensus on an extensive list of items. In the absence of a universally agreed method for defining and determining consensus [46, 47], we chose *a priori* a level of agreement or disagreement that we felt reflected a majority response, and was in line with similar research [48]. Most items achieved even higher levels of consensus (≥ 80%), but we acknowledge that defining and determining consensus differently may have altered the findings. The non-probability sampling methods used in Delphi surveys enable the targeting of participants with relevant knowledge, expertise and interest [19, 47], but limits generalisability. While we recruited professionals from two sources, selection bias cannot be excluded. Participants recruited through the AROC news item may have influenced responses on items relating to AROC data collection, although almost all Australian inpatient rehabilitation centres contribute data to AROC. We did not recruit based on location; thus, some smaller Australian jurisdictions were not represented, and most responses likely reflect urban settings. Further, given RITH for reconditioning is not widely established, most responders would likely have answered from a more theoretical position, drawing from their experiences of inpatient and/or community rehabilitation services. Thus, caution in extrapolating findings is required, and results may reflect the uniqueness of the Australian health system. However, we believe the findings can usefully inform the planning of RITH for reconditioning services, particularly given limited published research. As this study focused on the views of health professionals, it is essential that the perspectives of patients, families and carers on RITH should be the subject of future research.

## Conclusions

We found strong support amongst survey participants for RITH as hospital substitution to be widely available for appropriate patients needing reconditioning. It was agreed that RITH programs would be attractive to providers if more patients could receive rehabilitation for the same financial outlay, or if the same patient outcomes could be achieved for less cost than comparable inpatient rehabilitation. Several items for which consensus was achieved may be important to the financial sustainability of RITH but require further research. These include the utilisation of telehealth/telerehabilitation; appropriate patient selection; careful budget management; and interdisciplinary team work where appropriate. Further studies on clinical outcomes and resource utilisation of home-based compared with inpatient rehabilitation are needed. Importantly, widespread implementation of RITH as hospital substitution also requires supportive legislative and payment systems, including mechanisms that promote the integration of primary care, and appropriate clinical governance. In recent times it has become clear that a rehabilitation response to the COVID-19 pandemic is necessary [17]. RITH for reconditioning as hospital substitution may thus have an expanded reach and assist the post-acute health care system well into the future [49].

## Supplementary Information

**Additional file 1.** Survey items where consensus was not achieved.

**Additional file 2.** Survey items using multiple choice or ranking. Percentage of participants selecting options.

## Data Availability

The data generated and analysed during the current study are available from the corresponding author on reasonable request and pending ethics committee approval.

## Abbreviations

AFRM: Australasian Faculty of Rehabilitation Medicine
AN-SNAP: Australian National Subacute and Non-Acute Patient
AROC: Australasian Rehabilitation Outcomes Centre
FIM: Functional Independence Measure
GP: General practitioner
MBS: Medicare Benefits Schedule
MDT: Multi-disciplinary team
PREM: Patient reported experience measure
PROM: Patient reported outcome measure
RITH: Rehabilitation in the home
