# Supplementary material for: Developing a model for rehabilitation in the home as hospital substitution for patients requiring reconditioning: a Delphi survey in Australia
Source: BMC Health Serv Res. 2023 Feb 3;23:113. doi: 10.1186/s12913-023-09068-5 (PMC9895972; doi:10.1186/s12913-023-09068-5)
Supplement: Supplementary file 1 — Additional file 1. Survey items where consensus was not achieved. [file 12913_2023_9068_MOESM1_ESM.docx]

**Additional File 1:** Survey items where consensus was not achieved^1^

| Item No. | Survey Item | Percentage  agreement | Delphi Round |
| --- | --- | --- | --- |
| **STEP 1 Initial patient identification** | | | |
| 1 | Members of acute care teams are able to identify patients who might be suitable for RITH. | 64.1 | 1 |
| **STEP 2 Determining patient eligibility** | | | |
| 2 | Members of acute care teams are able to determine a patient’s eligibility for RITH. | 47.7 | 1 |
| 3 | The acute care physician or surgeon must agree that the patient is appropriate for RITH. | 59.2 | 1 |
| 4 | To be eligible for a RITH program, patients must be assessed as needing at least two different rehabilitation therapy types. | 52.3 | 1 |
| 5 | In determining a patient’s eligibility for RITH, a home visit should be undertaken. | 59.2 | 1 |
| 6 | Patients who score 1 to 5 on the Rockwood Clinical Frailty Scale (see scale illustration here) will be more suited to RITH than those who have higher degrees of frailty (i.e. who score 6 or above). | 63.4 | 2 |
| 7 | The patient is not deemed to be at high risk of having a fall. | 69.3 | 2 |
| 8 | RITH should be the default program for patients who are domestic ambulators only - i.e. able to manage at home during RITH, but cannot readily leave their homes to access the community. | 57.0 | 2 |
| 9 | Patients who can readily leave their homes (i.e., they have no access issues and have the ready availability of transport) should not have RITH, but rather have outpatient/clinic/day hospital rehabilitation. | 47.5 | 2 |
| **STEP 3 Development of the RITH care plan** | | | |
| 10 | At least two members (from different disciplines) of a rehabilitation service should assess the patient before the initial RITH care plan is developed. | 63.1 | 1 |
| 11 | Under Model 1 the RITH service should manage all of the patient’s general medical care throughout RITH (as would be the case during an inpatient rehabilitation episode). | 55.6 | 2 |
| 12 | Under Model 2 the RITH service should manage all of the patient’s general medical care throughout RITH (as would be the case during an inpatient rehabilitation episode). | 40.4 | 2 |
| 13 | If a patient is deemed to only need one allied health discipline type, then that service should not be classified as “RITH as hospital substitution”. | 39.6 | 2 |
| 14 | Funding should be the primary determinant of the intensity and duration of a RITH program. | 15.5 | 1 |
| 15 | A patient’s individual RITH program should be limited by a predetermined budget. | 19.7 | 1 |
| 16 | A patient’s individual RITH program should be limited by a predetermined maximum number of occasions of service. | 23.6 | 1 |
| 17 | A patient’s individual RITH program should be limited by a predetermined maximum duration. | 36.2 | 1 |
| 18 | The RITH program depends on a patient’s GP to provide ongoing general medical care during their participation in RITH. | 68.2 | 1 |
| 19 | There should be a written agreement between the patient’s GP and the RITH team, outlining the responsibilities of each during the RITH program. | 58.1 | 1 |
| 20 | The patient’s GP should be offered some form of an incentive payment as part of the RITH program to foster their engagement in RITH. | 38.4 | 2 |
| 21 | The employment or contracting of a general practitioner/GP service by a RITH provider is one solution for providing general medical care, where the patient’s own GP is unwilling or unavailable. | 54.5 | 2 |
| 22 | The primary responsibility for oversight and management of ongoing general medical care of the patient during RITH remains with the patient’s general practitioner. | 60.2 | 1 |
| 23 | The primary responsibility for oversight and management of ongoing general medical care of the patient during RITH does not sit with the rehabilitation physician. | 48.4 | 1 |
| 24, 25: What do you think are important components of a RITH case manager’s role? | | | |
|  | 24 - Facilitating equipment purchases required for the RITH program for the patient. | 69.0 | 1 |
|  | 25 - Managing the patient’s individual RITH budget allowance. | 66.7 | 1 |
| 26 | It is best if the case manager is not also delivering RITH therapy to the patient. | 37.6 | 2 |
| 27 | A rehabilitation nurse is ideally suited for a case management role because of their understanding of rehabilitation processes and clinical issues. | 54.5 | 2 |
| **STEP 4 RITH program delivery** | | | |
| 23 | A multi-disciplinary team member should be in the patient’s home to assist during any telehealth/telerehabilitation session with a rehabilitation physician. | 41.9 | 1 |
| 29 | Telehealth/telerehabilitation with the rehabilitation physician should only be used for progress reviews and other interactions with the patient where a physical examination of the patient is not required. | 66.0 | 2 |
| 30 | An MBS-approved GP case conference with members of the RITH team early in the RITH program is ideal. | 65.9 | 1 |
| 31 | The RITH program depends on a patient’s GP to provide ongoing general medical care during their participation in RITH. | 68.2 | 1 |
| **Budgetary Factors** | | | |
| 32 | The patient’s RITH care plan should include an indicative budget. | 43.1 | 1 |
| 33 | A schedule of costs (that is, a pricing schedule or pricing tool) for each type and mode of delivery of therapy interventions should be used to aid development of the patient’s RITH care plan. | 43.8 | 1 |
| 34 | It is not important to adhere to a patient’s individual RITH budget as long as the overall budget available for the whole RITH service is not exceeded. | 50.4 | 1 |
| 35 | The patient’s RITH rehabilitation physician should be responsible for overseeing each patient’s individual RITH program budget. | 22.0 | 1 |
| 36 | Only patients who are likely to have a successful RITH program (i.e. meet their goals) within the budget allocated for RITH should be offered RITH, otherwise they should have inpatient rehabilitation. | 50.0 | 2 |
| 37 | Where the patient’s clinical and support needs are likely to exceed the budget available for a RITH program, then that patient should not be offered RITH but have inpatient rehabilitation. | 58.0 | 2 |
| **Miscellaneous** | | | |
| 38 | There should be greater emphasis on providing sufficient inpatient rehabilitation capacity than to develop RITH for patients requiring reconditioning. | 20.5 | 1 |

^1^Table shows survey item, the percentage of participants agreeing with statement, and the Delphi round in which survey item appeared.
