# Supplementary material for: Developing a model for rehabilitation in the home as hospital substitution for patients requiring reconditioning: a Delphi survey in Australia
Source: BMC Health Serv Res. 2023 Feb 3;23:113. doi: 10.1186/s12913-023-09068-5 (PMC9895972; doi:10.1186/s12913-023-09068-5)
Supplement: Supplementary file 2 — Additional file 2. Survey items using multiple choice or ranking. Percentage of participants selecting options. [file 12913_2023_9068_MOESM2_ESM.docx]

**Additional File 2:** Survey items using multiple choice or ranking. Percentage of participants selecting options.

| Item No. | Question | Percentage | Delphi Round |
| --- | --- | --- | --- |
| **Development of the RITH care plan** | | | |
| 1 | When is the best time for the development of the RITH care plan?  Please rank the following options in order of priority from (1) best choice to (3) least-best choice. | Ranked (1) | 1 |
|  | - Before the patient is discharged from acute care. | 63.8 |  |
|  | - As soon as possible after the patient is discharged home. | 13.8 |  |
|  | - Following the first multidisciplinary case conference | 25.4 |  |
| **RITH program delivery** | |  |  |
| 2 | Who do you think the case manager should be for a RITH patient?  Please rank the following options in order of priority from (1) best choice to (5) least-best choice. | Ranked (1) | 1 |
|  | - The patient’s RITH rehabilitation physician. | 3.2 |  |
|  | - A member of the RITH multi-disciplinary team that is treating the patient | 60.3 |  |
|  | - An independent RITH team member (that is, someone who is not treating the patient) | 30.2 |  |
|  | - Someone who is independent of the RITH program | 2.4 |  |
|  | - No case manager is needed | 6.4 |  |
| 3 | How people work together in teams is an important aspect of the delivery of patient care. This question asks you to consider a **multidisciplinary** versus an **interdisciplinary** approach. **Multidisciplinary teamwork -** where therapists work in an integrated manner, but in parallel with each other. **Interdisciplinary teamwork -** where therapists work in an integrated manner and strive to do cross disciplinary work wherever possible (but still within their scope of practice). Which model do you prefer for RITH? |  | 2 |
|  | - Multidisciplinary | 37.0 |  |
|  | - Interdisciplinary | 57.0 |  |
|  | - Unsure | 6.0 |  |
| 4 | Two different models for RITH will now be considered. Model 1 is where RITH mirrors the equivalent inpatient rehabilitation episode in terms of duration and intensity. Model 2 is where RITH could be longer than the equivalent inpatient episode, but of less intensity. Overall, let’s assume that the resources required to deliver Model 1 and Model 2 would be similar. What would be your preference? |  | 2 |
|  | - Model 1 | 36.4 |  |
|  | - Model 2 | 47.5 |  |
|  | - Undecided | 16.2 |  |
| 5 | What do you think is the maximum length of time a RITH service should be able to offer a RITH program for?  *Please assume that* ***therapy intensity*** *is able to be varied across an individual patient's RITH program (e.g., short and intense, long and less intense, or intensity weighted towards the early phase), but that the* ***overall*** *resources required will be similar irrespective of program length.* |  | 2 |
|  | - Up to 3wks | 10.0 |  |
|  | - Up to 6wks | 33.0 |  |
|  | - Up to 10wks | 37.0 |  |
|  | - Other | 20.0 |  |
| **RITH Program Discharge** | | | |
| 6 | As a key performance indicator for RITH, what do you think is an acceptable rate of a subsequent admission to inpatient rehabilitation following a ‘failed’ RITH for reconditioning program? |  | 2 |
|  | - Less than 5% | 32.3 |  |
|  | - 5-10% | 46.5 |  |
|  | - 11-20% | 19.2 |  |
|  | - More than 20% | 2.0 |  |
| **Clinical governance** | | | |
| 7 | At what point should a rehabilitation physician first become involved in the care of a patient for whom a RITH program is being considered or has commenced? Please rank the following options in order of priority from (1) best choice to (4) least-best choice. | Ranked (1) | 1 |
|  | - While the patient is in acute care | 75.2 |  |
|  | - Within a week of discharge from acute care | 15.0 |  |
|  | - Beyond one week of discharge from acute care | 1.7 |  |
|  | - It is not necessary for a rehabilitation physician to become involved in the care of a patient who is to have RITH | 10.6 |  |
| 8 | How should a rehabilitation physician be, or remain, involved in each patient's RITH program? Please rank the following options in order of priority from (1) best choice to (4) least-best choice. | Ranked (1) | 1 |
|  | - By attending case conference only | 11.7 |  |
|  | - By attending a regular individual weekly review (in person or by telehealth) of each patient, in addition to attending case conference | 48.8 |  |
|  | - By attending a less frequent individual review (in person or by telehealth) of each patient, in addition to attending case conference | 32.2 |  |
|  | - It is not necessary for a rehabilitation physician to review a patient in person or by telehealth as part of their RITH program | 12.6 |  |
